# Response to Crespo *et al.* Comments on: Varela-Mato, V.; *et al.* Lifestyle and Health among Spanish University Students: Differences by Gender and Academic Discipline. *Int. J. Environ. Res. Public Health* 2012, *9*, 2728–2741

**DOI:** 10.3390/ijerph10083593

**Published:** 2013-08-13

**Authors:** Veronica Varela-Mato

**Affiliations:** Faculty of Education and Sports Science, University of Vigo, Xunqueira Campus, Pontevedra 36005, Spain; E-Mail: V.Varela-Mato@lboro.ac.uk

We thank Dr. Crespo [1] for his interest in reading our article and his time in writing his comments on work [2]. However, we have to respectfully point out our disagreement and a few comments of our own:

**Comment:** The authors [2] transfer their results to the Spanish university with a transversal descriptive study without selection of the participants and that includes 27% of the students in one of the three campuses and does not exceed 5% of total Students at the University of Vigo, and in which nothing is known of the subjects who did not respond to the questionnaires (see “The STROBE Statement”) [3].

**In response to this comment, I suggest to look at the limitations of the article:** “First, relying on cross-sectional data constrains the study’s ability to make causal inferences about the relationship found in the research. Secondly, the study sample was not randomly selected, and it may have not been representative of all the university students of the region in which the research was carried out, since only those who attended class were who answered the questionnaires. Therefore, caution needs to be exercised when attempting to generalize to other contexts or populations.”

**Comment:** The authors use the short version of the International Physical Activity Questionnaire (IPAQ [4]) questionnaire and defined as sufficiently active to those who perform an energy expenditure greater than or equal to 1,500 Met-min/week. IPAQ itself [4] and related agencies such as the U.S. Department of Health and Human Services (USDHHS [5]), the UK National Institute of Health (NICE [6]), and the World Health Organization (WHO [7]), indicate that it is from an energy expenditure of 500 Met-min/week when defining a person as sufficiently active (equivalent to performing 150 min/week of moderate physical activity or 75 min/week vigorous physical activity).

**In regards to this comment,** we would like to clarify that in our article we based our discussion in this reference [8], which differentiates between minimally active and health-enhancing PA levels:

To be active and achieve Health-enhancing (HEPA) PA levels the following is required: vigorous activity ≥ 3 d/week, totaling ≥1,500 Met-min/week, or ≥7 d/week of any combination of walking, moderate-intensity, or vigorous activities, totaling > 3,000 Met-min/week. 

To be minimally active but without achieving HEPA: ≥3 d/week ofvigorous activity of ≥20 min/d, or ≥5 d/week ofmoderate-intensity activity or walking ≥ 30 min/d, or ≥5 d/week ofany combination of walking, moderate-intensity, or vigorous activities, totaling ≥ 600 Met-min/week.

Therefore to achieve Health-enhancing PA and be active ≥1,500 Met-min/week of vigorous activity or >3,000 Met-min/week of a combination of low, moderate and vigorous should be achieved. For this reason we state: “It has been found that most of the students did not reach those levels established by the recommendations guidelines for health-enhancing PA (value of 1,500 Met-min/week) [1].”

**Comment:** With respect to the claim that the Varela-Mato *et al.* research. was approved by the Ethics Committee of the University of Vigo, this cannot be because in that there is no University Research Ethics Committee and there is only one ethics committee for the welfare of laboratory animals (SIUV [9]).

**Response:** In response to this, the authors have written a correction letter [10] regarding this matter to avoid future comments regarding the same topic.
